# Metabolomic Strategies in Biomarker Research–New Approach for Indirect Identification of Drug Consumption and Sample Manipulation in Clinical and Forensic Toxicology?

**DOI:** 10.3389/fchem.2019.00319

**Published:** 2019-05-10

**Authors:** Andrea E. Steuer, Lana Brockbals, Thomas Kraemer

**Affiliations:** Department of Forensic Pharmacology and Toxicology, Zurich Institute of Forensic Medicine, University of Zurich, Zurich, Switzerland

**Keywords:** metabolomics, biomarker, NPS, drugs of abuse, urine adulteration, indirect, metabolism

## Abstract

Drug of abuse (DOA) consumption is a growing problem worldwide, particularly with increasing numbers of new psychoactive substances (NPS) entering the drug market. Generally, little information on their adverse effects and toxicity are available. The direct detection and identification of NPS is an analytical challenge due to their ephemerality on the drug scene. An approach that does not directly focus on the structural detection of an analyte or its metabolites, would be beneficial for this complex analytical scenario and the development of alternative screening methods could help to provide fast response on suspected NPS consumption. A metabolomics approach might represent such an alternative strategy for the identification of biomarkers for different questions in DOA testing. Metabolomics is the monitoring of changes in small (endogenous) molecules (<1,000 Da) in response to a certain stimulus, e.g., DOA consumption. For this review, a literature search targeting “metabolomics” and different DOAs or NPS was conducted. Thereby, different applications of metabolomic strategies in biomarker research for DOA identification were identified: (a) as an additional tool for metabolism studies bearing the major advantage that particularly a priori unknown or unexpected metabolites can be identified; and (b) for identification of endogenous biomarker or metabolite patterns, e.g., for synthetic cannabinoids or also to indirectly detect urine manipulation attempts by chemical adulteration or replacement with artificial urine samples. The majority of the currently available literature in that field, however, deals with metabolomic studies for DOAs to better assess their acute or chronic effects or to find biomarkers for drug addiction and tolerance. Certain changes in endogenous compounds are detected for all studied DOAs, but often similar compounds/pathways are influenced. When evaluating these studies with regard to possible biomarkers for drug consumption, the observed changes appear, albeit statistically significant, too small to reliably work as biomarker for drug consumption. Further, different drugs were shown to affect the same pathways. In conclusion, metabolomic approaches possess potential for detection of biomarkers indicating drug consumption. More studies, including more sensitive targeted analyses, multi-variant statistical models or deep-learning approaches are needed to fully explore the potential of omics science in DOA testing.

## Current Challenges in Analytical (Forensic) Toxicology

Forensic toxicology is a field of science dedicated to the application of accepted and validated scientific methods and practices in toxicology to cases and issues where drug effects may have administrative or medico-legal consequences, and where the results are likely to be used in court (The Forensic Toxicology Council, 2010). Main questions are related to behavioral or human performance toxicology such as impaired driving assessment or drug facilitated crimes, postmortem toxicology, abstinence control or workplace drug testing (Wyman, 2012). Primarily, forensic toxicology encompasses the qualitative and quantitative analysis of ethanol, drugs of abuse (DOA), prescription drugs or poisons in biological matrices, mainly blood or urine, and the interpretation of the respective results. Commonly, routine laboratory procedures include the use of prescreen immunoassays (IA) to test for the most relevant DOAs—often followed by confirmatory analyses such as hyphenated chromatographic techniques; e.g., gas chromatography (GC)—mass spectrometry (MS) or liquid chromatography (LC)-MS (Drummer, 2007; Maurer, 2007, 2010). Furthermore, so-called general unknown screening approaches using GC-MS, LC-MS/(MS) or LC-high resolution (HR) MS are applied. In order to assess the level of exposure, positive results from the qualitative screening analyses are subsequently confirmed and quantified, if the compounds were found to have relevant toxic potential. With recent developments on the (il)legal drug market, such as the constant appearance of new psychoactive substances (NPS) or easily available drug masking agents and procedures, the field of forensic toxicology currently faces a variety of new challenges.

More than 800 NPS have been reported to the United Nations Office on Drugs and Crimes (UNODC) Early Warning Advisory as of December 2017, making their use and misuse a global problem [United Nations Office on Drugs and Crime (UNODC) (2018)]. Of these, 68% were synthetic cannabinoids and stimulants, which make up the largest fraction of newly reported NPS in 2017. Generally, little information on the adverse effects and toxicity of NPS are available posing a growing problem worldwide. In addition, their direct detection and identification remains an analytical challenge due to their ephemerality on the drug scene. Common IAs are usually unable to reliably pick up whole classes of NPS which makes the development of comprehensive screening approaches mandatory for their detection. While being very sensitive, targeted methods applying e.g., multiple reaction monitoring (MRM) constantly need to be updated and require reference standards that are often missing or are associated with high costs. HRMS has shown strong potential, as the need for method adjustment is omitted and it allows for retrospective data evaluation (Grabenauer et al., 2012; Shanks et al., 2012). Accurate mass facilitates compound identification, but the use of HRMS instruments leads to limitations concerning sensitivity and dynamic range. Further, data processing of HR data for unknowns is still very laborious and time consuming.

An alternative approach would be the development of novel screening methods, that are not directly targeting the analyte's or its metabolite's chemical structures. This could be highly beneficial to provide fast response on suspected NPS consumption and aid in the overall resolution of this complex analytical scenario. A first approach was presented in a recent work from Cannaert et al. showing that it is possible to develop an assay that detects synthetic cannabinoids and their metabolites based on their activity and interaction with the cannabinoid receptors (Cannaert et al., 2017). Such an activity-based screening assay might complement conventional analytical methods (targeted and untargeted) and serve as a front-line screening tool of urine. Despite the fact that it is impossible to positively identify specific synthetic cannabinoids with this approach, it potentially reduces the number of false negative results compared to targeted approaches, where a compound is missed if not included in the candidate list (Bijlsma et al., 2018).

The incentive for drug users to switch to NPS is often not solely based on getting high legally, but potentially also to circumvent positive results in drug-screening tests. The latter is particularly relevant, where drug abstinence needs to be proven, e.g., driving liability testing, certain psychiatry or prison settings or in workplace drug testing procedures (EMCDDA, 2009; Bijlsma et al., 2018). In abstinence control settings, urine still represents the matrix of choice (Verstraete, 2004; Phan et al., 2012; Fu et al., 2014). Hence, it is critical for laboratories to detect urine adulteration attempts that might aim at circumventing positive drug testing results (Wu et al., 1999). Common manipulation procedures involve dilution of authentic urine, substitution with artificial urine or chemical adulteration. The use of a variety of different chemicals has been reported due to their known masking effects during drug detection. Generally applicable for chemical urine adulteration are common household chemicals such as peroxidase and peroxide (H_2_O_2_), hypochlorite-based bleach (NaOCl) or pyridinum chlorochromate (PCC), potassium nitrite (KNO_2_), and iodine (I_2_) (Uebel and Wium, 2002; Jaffee et al., 2007; Fu et al., 2014). Commercialized products for urine adulteration are particularly prevalent in the US, where products such as Stealth® (containing peroxidase and H_2_O_2_) (Valtier and Cody, 2002), Klear® (containing KNO_2_) (Peace and Tarnai, 2002) or Whizzies® (containing sodium nitrite) (Dasgupta et al., 2004) and “Urine Luck” (containing PCC) (Wu et al., 1999; Paul et al., 2000) are readily available via the internet (Dasgupta, 2007; Jaffee et al., 2007; Fu et al., 2014). The same applies for commercially available artificial urine products (Goggin et al., 2017; Kluge et al., 2018). While it should be mandatory for toxicological laboratories to screen for the large selection of chemical adulterants and artificial urine products, time, costs, and resources often prevent comprehensive testing. A time- and cost-effective alternative are spot and dipstick tests, integrity testing or integrated sample checks to commercially available IA systems. However, these are often associated with high rates of false negative or false positive results (Edwards et al., 1993; Fu et al., 2014; Matriciani et al., 2018). Ideally, a drug testing workflow would include drug detection with simultaneous screening for adulteration attempts within the very same run, in particular during high-throughput testing.

To cope with these recent developments, methods which are not focused directly on the analyte or adulterant in question are an attractive approach. Next to the described indirect activity-based screening approach, the application of metabolomics or metabolomics-related techniques (applying common metabolomics data analysis and statistics) represents such an alternative strategy for the identification of biomarkers useful for the (indirect) detection of drug consumption or manipulation attempts. The aim of the present review is to summarize available data on the search of potential biomarkers for drug consumption and sample adulteration as well as their interpretation utilizing metabolomics approaches. Therefore, a PubMed search has been conducted targeting “metabolomics” along with different DOAs or NPS or urine adulteration.

## Metabolomics

**Figure 1:**
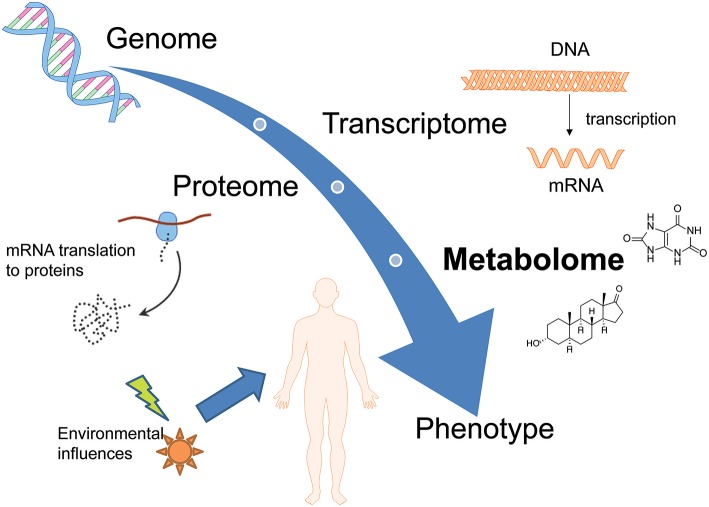
Overview of different omics-sciences such as genomics, transcriptomics, and proteomics. Metabolomics represents the downstream output of the genome but also the upstream input from the environment and is therefore positioned at the bottom of the “omics” cascade.

Metabolomics (also known as metabolic profiling or metabonomics) is the study of the metabolism and metabolites in an organism and is one of many “omics” sciences such as exposomics (the study of the complete collection of environmental exposures), microbiomics (the study of the microbiome), proteomics, genomics, and transcriptomics. Metabolome studies target the qualitative and quantitative characterization of small molecules (<1,000 Da) with changes appearing in organisms in response to a certain stimulus. The metabolome is unique, dynamic and related to the phenotype (Dinis-Oliveira, 2014; Wishart, 2016). In contrast to the other “omics”-sciences, metabolomics is able to link both gene and environmental interactions. It not only represents the downstream output of the genome but also the upstream input from the environment and is therefore positioned at the bottom of the “omics” cascade as represented in Figure 1 (Wishart, 2016; Zaitsu et al., 2016). In recent years, metabolomics approaches were applied to various fields, due to the ability to detect subtle changes in a large dataset with comprehensive metabolite measurements. The question which metabolites are actually considered to be part of the “metabolome” is still controversial, resulting in partly confusing definitions. The metabolites present in biological systems and defined as the metabolome in the strict sense, include endogenously derived biochemicals e.g., carbohydrates, lipids, amino acids, fatty acids, steroids, or vitamins. However, metabolomic analyses also allow for the detection of exogenously derived metabolites from xenobiotics and/or their phase I and phase II metabolites; this can be referred to as the xenometabolome (David et al., 2014). Particularly difficult is, however, the differentiation of studies focusing on (xenobiotic) drug metabolism, without applying metabolomics techniques. For example, a study published by Patton et al. is named “Targeted Metabolomic Approach for Assessing Human Synthetic Cannabinoid Exposure and Pharmacology” but is actually focusing on conventional approaches for chiral analysis of JWH-018 and AM2201 metabolites (Patton et al., 2013). Some recent review articles on different drugs—also entitled “metabolomics of” actually contain more information on the drug's metabolism and pharmacodynamics than changes of the metabolome as defined above (Dinis-Oliveira, 2016a,b). To avoid confusion, the following review will focus on either metabolomic studies targeting endogenous compounds or applying metabolomic techniques and statistics for the elucidation of the xenometabolome.

In general, metabolomics is a valuable tool in different disciplines such as drug discovery (Lu and Chen, 2017; Mercier et al., 2018), biomarker research (Klein and Shearer, 2016; Wang et al., 2016b; Zhang et al., 2016; Ambati et al., 2017), studies of diseases (Klein and Shearer, 2016; Ren et al., 2016; Wurtz et al., 2016), and metabolic pathways confirmation (Ren et al., 2016; Zhang et al., 2016). It involves both the identification of endogenous substances in different biological samples as well as the statistical analysis of differences between two or more conditions. Fields in which metabolomics studies have previously been reported include clinical trials, toxicology, pharmacology, and nutrition (Brignardello et al., 2017; Korsholm et al., 2017; Cornelis et al., 2018; Wu et al., 2018). However, within the field of forensic (toxicology) this approach is rather new, with little human data available so far. Nevertheless, metabolomics approaches in forensics become more and more popular as they were found to be a helpful tool for a variety of forensic questions, e.g., in postmortem investigations (Castillo-Peinado and Luque de Castro, 2016). For the estimation of the postmortem interval (PMI), metabolomics studies found elevated levels of amino acids and creatinine postmortem (Castillo-Peinado and Luque de Castro, 2016) and decreasing levels of sterol sulfates and very-long-chain fatty acids within the postmortem period (Wood and Shirley, 2014). Additionally, biomarker research within the field of forensic toxicology might successfully be used to investigate consumption behavior, to distinguish between acute or chronic drug consumption or to find the underlying mode of toxicological action in humans (Wang et al., 2016a). Thereby, different applications of metabolomics strategies in biomarker research for DOA identification were proposed: (a) as an additional tool for metabolism studies bearing the major advantage that particularly a priori unknown or unexpected metabolites can be identified; and (b) for identification of endogenous biomarker or metabolite patterns, e.g., for synthetic cannabinoids or also to indirectly detect urine manipulation attempts such as artificial urine samples or chemical adulteration. The majority of the currently available literature deals with metabolomic studies for DOAs to better assess their acute or chronic effects or to find biomarkers for drug addiction and tolerance.

### Analytical Techniques in Metabolomics

**Figure 2:**
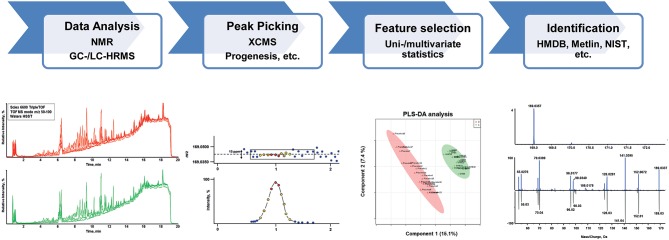
Schematic of a typical untargeted metabolomics workflow including data analysis, feature detection (peak picking), statistical evaluation and compound identification.

In principle, two major kinds of metabolomic analyses can be applied—targeted and untargeted—which are in detail reviewed elsewhere (Chen et al., 2007; Dettmer et al., 2007; Dunn, 2011; Monteiro et al., 2013; Zhang et al., 2016; Cuykx et al., 2018; Ghanbari and Sumner, 2018; Kind et al., 2018). While targeted analysis will focus on an a priori known number of defined metabolites, untargeted metabolomics or discovery metabolomics aims to capture all metabolomic information in a sample. In the latter, features of interest are filtered after data acquisition applying different uni- and multi-variate statistical methods followed by their identification. A schematic of an untargeted metabolomics workflow, the method of choice for biomarker search, is given in Figure 2. A wide variety of targeted and untargeted methods have already been reported in the literature for the separation and quantification of components belonging to the metabolome. However, it was found that no single analytical platform is capable of capturing all metabolomics information in a single run (Dinis-Oliveira, 2014). LC- and GC-MS, nuclear magnetic resonance (NMR) spectroscopy, and LC with electrochemical detection are all used (Ning et al., 2018), but the most widespread analytical instruments utilized are MS and NMR spectroscopy. The advantages and disadvantages of MS or NMR use within the field of metabolomics have been extensively discussed in corresponding reviews (Schlotterbeck et al., 2006; Pan and Raftery, 2007). In summary, MS shows much better sensitivity and resolution and the ability for high-throughput applications, while NMR profits from a comprehensive coverage of chemical species (Chen et al., 2007). NMR analysis certainly provides several advantages in metabolome studies, however, GC- and LC-MS platforms are more widely available in forensic toxicological laboratories (Drummer, 2007; Maurer, 2010; Peters, 2011; Meyer et al., 2014). For introducing a prepared biological sample into a mass spectrometer, GC, LC, direct injection or capillary electrophoresis can be used (Dettmer et al., 2007). In recent years, LC-MS techniques gained importance having the advantage of simpler sample preparation approaches compared to e.g., GC techniques where one- or two-step derivatization is usually mandatory. Avoiding numerous, tedious sample preparation steps can reduce overall measurement variations and will result in more reliable and comparable metabolomics data. Reversed-phase (RP) methods using C18 stationary phases in combination with mobile phases consisting of water (A) and methanol or acetonitrile (B), with additional formic acid (FA), are often the preferred choice due to their non-specific retention mechanism. Although this method is powerful in separating many—especially lipophilic metabolites such as steroids (Marcos et al., 2014) or endocannabinoids (Pastor et al., 2014), use of this single platform is non-optimal, lacking retention for polar metabolites and resolution for many apolar metabolites. Hydrophilic liquid interaction chromatography (HILIC), capillary electrophoresis, and ion-pairing reversed-phase chromatography are solutions often described in metabolomic applications to increase the separation of polar metabolites (Cuykx et al., 2018). For targeted analysis, all kind of MS devices, including triple quadrupole instruments, can generally be applied. For untargeted screening approaches, MS instruments with high-resolution mass measurements, such as time-of-flight (TOF), quadrupole TOF (qTOF) or Fourier transformation (FT) e.g., Orbitrap mass spectrometers, are preferred. Generally, some kind of MS/MS data acquisition, mostly based on data-dependent acquisition (DDA), is used to generate further MS information for identification. Data independent acquisition can increase totality of MS/MS information, but at present cannot be handled by many (commercial) software solutions for data analysis (Boxler et al., 2018a). Knowledge of accurate masses provides the basis for peak identification across different samples as it allows for calculation of empirical formulae and facilitates feature identification using online or commercially available databases such as METLIN (Guijas et al., 2018), the Human Metabolome Database (Wishart et al., 2007) (HMDB, V4.0), NIST (Linstrom and Mallard, 2001), and Lipidblast (Kind et al., 2013) as well as a priori unknown identification.

### Quality Control and Compensation of Variations in Untargeted Metabolomics

If no reference material is available for the metabolites of interest—as will be the case in untargeted metabolome studies—comparison between groups and/or conditions will be performed on the basis of relative abundances. Reproducible measurements thereby are a prerequisite for reliable data processing and analysis. It is evident, that method validation in the classical sense is impossible for untargeted metabolomics. Nevertheless, quality control (QC) is essential and needs to be implemented (Dunn et al., 2012; Cuykx et al., 2018). Different strategies—at best in combination—are generally accepted: addition of internal standards (IS) into each analyzed sample; continuous measurement of system suitability tests (SST) over the whole analytical batch containing a defined number of metabolites ideally evenly distributed over the chromatographic run; and inclusion of QC pool samples prepared from all experimental samples, hence representing the average of the data set (Dunn et al., 2012; Broadhurst et al., 2018). Further, randomization of the samples at least for analysis is an important step in untargeted metabolomics to prevent technical/instrumental biases (Dunn et al., 2012).

Typically, all samples of one metabolome experiment—ideally collected and stored under the same controlled conditions—will be measured within the same batch in order to avoid bias caused by sampling, storage or day to day variations in instrument performance. Recently, Nielsen et al. performed an untargeted metabolome experiment on a retrospective dataset collected for forensic toxicology routine analysis. There, a subgroup of samples positive for 3,4-methylenedioxymethamphetamine (MDMA) was compared to a negative control group. Only small retention time shifts were observed across all sample chromatograms, despite the fact that data was collected over a long period of time. Normalization by the NOMIS method which utilizes variability information from multiple IS compounds to find an optimal normalization factor for each individual feature (Sysi-Aho et al., 2007), also enabled the distinction between biological variation from obscuring variation related to sample preparation and ion source variation. The fact that MDMA and its phase I and II metabolites were positively identified and clearly up-regulated in MDMA users (specified by almost all applied methods) proved successful overall data-analysis. These findings suggest that analysis of retrospective data is generally possible, e.g., if samples are initially collected in the same sampling tubes, sample storage and preparation is highly standardized, and routine methods are quality controlled as is typically the case in routine forensic analysis (Nielsen et al., 2016). Mollerup et al. successfully applied a similar strategy to search for new markers/metabolites of valproic acid ingestion amenable to positive electrospray ionization (ESI) (Mollerup et al., 2019).

### Data Processing and Statistics in Untargeted Metabolomics

Metabolomics platforms generate large and complex data highlighting the need for appropriate data processing tools that allow the preparation of chromatographic and spectral data for multivariate data analysis (Katajamaa and Oresic, 2007). Often procedures like data condensation and reduction (by means of centroiding and deisotoping mass spectra), chromatographic alignment (to prevent misinterpretations due to retention time variations), filtering (for removal of noise or background signals) and peak recognition, and collection [by applying threshold windows for mass (m/z) and retention time] are used in this context. Details on different options of data processing can be found e.g., in a recent publication of Cuykx et al. (2018). To achieve minimal influence of systematic and sample biases (e.g., degree of urine dilution), it is recommended to normalize MS data either by the parameters of the whole dataset (e.g., total ion count, median ion count, etc.) or by the intensities of multiple or single ISs (Sysi-Aho et al., 2007). Data of biological origin is often skewed and commonly quantitative data are mean-centered, log-transformed, and normalized (unless absolute quantification is carried out). As every data processing step (for example filtering, scaling, peak picking, missing value imputations, and normalization) can have a significant impact on the interpretation of experimental results, it needs to be adequately described in the method sections (Yin and Xu, 2014; Alonso et al., 2015).

Different kinds of statistical tests—uni- and multi-variate—are usually performed for data interpretation. Univariate tests (*t-*test; ANOVA) are straightforward and compare the intensities of single features between different groups. While the interpretation of these tests is very clear, the need for multiple repetitions for hundreds of variables in metabolomic studies increases the risk for detection of false positive features (Broadhurst and Kell, 2006; Kim and van de Wiel, 2008). This should ideally be accounted for by applying false discovery corrections (e.g., Bonferroni or Benjamini–Hochberg correction). The most widely used unsupervised multivariate technique in science is principal component analysis (PCA). It projects the maximum variance of a multi-dimensional space in principal components and summarizes the data set in a limited number of components. PCA is mainly used as an exploratory technique as it is unsupervised and hence does not explicitly account for class-based separations. In contrast to this, (orthogonal) partial least-square discriminant analysis [(O)PLS-DA] is a supervised multivariate technique, which describes the greatest variance to differentiate between experimental classes, with the aim to find the metabolic patterns that are most important for the classification. Based on S-plots or variable importance in projection (VIP) values, the metabolites that have a large impact on the projection are selected. Supervised multivariate approaches are prone to overfitting to irrelevant or noisy features. Therefore, cross-validation procedures need to be carried out (Broadhurst and Kell, 2006; Gromski et al., 2014; Cuykx et al., 2018).

## Applications of Metabolomics for Forensic (Toxicology) Purposes

As already stated above, the steadily increasing number of NPS and general changes of the (il)legal drug market represent a major challenge for forensic toxicology laboratories. Applying metabolomics or metabolomics-related techniques might be a beneficial alternative strategy to overcome some of the resulting issues. In general, available literature on metabolomics in the field of forensic toxicology can be grouped into four different categories: (a) biomarker search by screening for new (exogenous) drug metabolites applying metabolomics techniques; (b) search for endogenous biomarkers indicative for acute drug intake or sample manipulation; or (c) search for endogenous biomarkers for addiction or to assess the severity of intoxications. A last group (d) includes studies that aim to elucidate the mechanisms of drug action, e.g., to improve existing or establish new therapy options. While the studies of the latter two groups were initially not designed to identify biomarkers potentially improving analytical detection in forensic toxicology, they might serve as a basis to further evaluate the general applicability of metabolome changes as biomarkers. In the following, these 4 groups are discussed in detail and a table for each group summarizes methodological and analytical characteristics of the references discussed.

### Metabolomics for Drug Metabolism Studies

Knowledge on a drug's biotransformation is of major importance for a variety of questions. For urine screening approaches, particularly if the parent compound itself remains undetectable in urine specimens, effective detection of drug intake will only be possible over one or several of its (unique) metabolites. Usually, focusing on highly abundant/major metabolites will be sufficient. However, in case of common major metabolites for several structurally related compounds, other minor metabolites might be necessary to finally prove intake of a particular drug. Also, if metabolism studies are performed in other than human species (e.g., rats or mice), investigation of minor metabolites in these studies is very important, as different excretion patterns in humans are likely. Hence, a minor metabolite in an animal study could become a major metabolite for human excretion. One major challenge in urine drug metabolism studies represents the definite identification of xenobiotic metabolites considering that urine contains a large variety of chemical species. Common strategies to identify metabolites involve the use of processing filters to exclude expected metabolites. For instance, typically occurring mass differences derived from prevalent, described metabolic reactions such as oxidation (+16) or demethylation (−14) can be extracted in a targeted manner. Additionally, thorough evaluation of MS/MS data or typical MS/MS patterns can be the basis for structure elucidation of new metabolites (Kim et al., 2018). However, the major disadvantage of these strategies is their restriction to a priori known, typical changes. More individual biotransformation e.g., dealkylation, hydrolysis, peroxidation or structural rearrangements are typically not covered (Guengerich, 2001; Chen et al., 2007; Kim et al., 2018). Untargeted metabolomics techniques were used as an alternative approach to detect new (uncommon) drug metabolites. A review by Chen et al. extensively describes and discusses the use of metabolomics in drug metabolism research (Chen et al., 2007). An overview on DOAs or compounds of forensic interest applying these techniques is given in Table 1.

**Table 1:** Summary of studies applying untargeted metabolomics approaches for the elucidation of xenobiotic drug metabolism.

**Parent compound**	**Newly identified metabolites**	**Experimental setup**	**Sample preparation**	**Analytical conditions**	**Data evaluation**	**Reference**
CBD	HO-CBD (3 isomers) Di-HO-CBD CBD oxidation 3″-carboxy-dinorCBD 2″-carboxy-trinorCBD	Untargeted Rat brain Placebo vs. CBD *n* = 5 per group	Homogenization SPE for phospholipid removal	LC-HRMS Poroshell 120 EC-C18 (100 × 3.0 mm, 2.7 μm) H_2_O, 0.1% FA ACN, 0.1% FA ESI pos/ne.g.,-qTOF Fullscan DDA auto MS/MS function	XCMS online Metaboanalyst 3.0	Citti et al., 2018
GHB	GHB-carnitine GHB-glycine GHB-glutamate	Untargeted Human urine Controlled administration Placebo vs. GHB Crossover design *n* = 19 per group Authentic samples *n* = 10	Dilution/filtration	LC-HRMS XSelect HSST RP-C18 (150 mm × 2.1 mm, 2.5 μm) 10 mM NH_4_COOH, 0.1% FA MeOH, 0.1% FA Merck SeQuant ZIC HILIC (150 mm × 2.1 mm, 3.5 μm) 25 mM NH_4_Ac, 0.1% HOAc ACN, 0.1% HOAc ESI pos/neg-qTOF Fullscan Additional run in MS/MS mode (DDA)	Progensis Qi Metaboanalyst 4.0	Steuer et al., 2018c
Sildenafil	Reduced sildenafil Deethylation/oxidation Deethylation/ demethanamine Demethylation/oxidation Demethylation/oxidation Mono-oxidation	untargeted Human liver microsomes With/without cosubstrates *n* = 3 per group	PP	LC-HRMS Kinetex^®^ C18 (150 × 2.1 mm, 2.6 μm) H_2_O, 0.1% FA ACN, 0.1% FA ESI pos/Orbitrap Fullscan DDA MS/MS	MZmine 2 SIMCA 14.0 STATISTICA 7.0	Kim et al., 2018
Valproic acid	3-hydroxy-4-en-valproic acid Valproylcarnitine 6 unidentified metabolites	Untargeted Human whole blood Exploration data set *n* = 68 (28% valproic acid pos) Test set *N* = 37 (32% valproic acid pos)	PP	LC-HRMS Acquity HSS C18 (150 × 2.1 mm, 1.8 μm) 5 mM NH_4_COOH, FA (pH 3) ACN, 0.1% FA ESI pos /qTOF MS^E^ mode (DIA)	UNIFI Python 3.6	Mollerup et al., 2019

**Figure 3:**
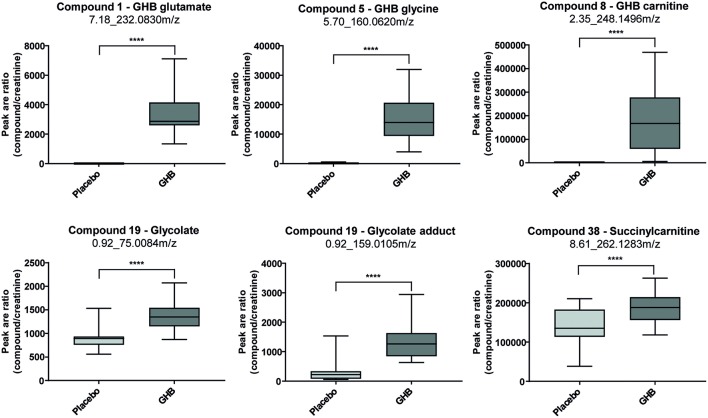
Box plots for promising analytical targets of GHB consumption representing observed changes between placebo and GHB intake [shown as analyte peak area to creatinine peak area ratios (*n* = 19 each)]. Statistical evaluation was carried out using a paired *t*-test (*p* < 0.05; ****p < 0.0001). Reprinted (adapted) with permission from Steuer et al. (2018c). Copyright 2018, Wiley.

Steuer et al. used an untargeted metabolome approach to search for new biomarkers of gamma-hydroxybutyric acid (GHB) intake. GHB's use as a knockout drug in cases of drug facilitated crimes makes it particularly important in forensic toxicology. The detection of GHB and particularly differentiation between exogenous intake and endogenous base levels remain challenging because of its extremely short detection windows (only up to 12 h in urine) caused by a fast metabolism. In contrast to many other DOAs, for GHB no metabolite has been identified so far, that allows for longer GHB detection compared to using the parent compound itself. Analysis of urine samples collected 4.5 h after GHB or placebo intake of a randomized, double-blind, placebo-controlled crossover study in 20 men allowed identification of novel GHB metabolites GHB carnitine, GHB glycine, and GHB glutamate as exemplified in Figure 3. However, more studies addressing quantitative values, pharmacokinetics, and stability are demanded for a final conclusion on the routine applicability of these markers (Steuer et al., 2018c). Mollerup et al. performed an interesting omics-based retrospective analysis to identify potential markers of valproic acid in blood that should allow the detection of valproic acid intake using the commonly applied positive ESI-MS mode. The antiepileptic drug valproic acid represents an important compound in forensic toxicological analysis, but can only be detected with negative ionization techniques or by GC-MS (Mollerup et al., 2019). A retrospective data evaluation of routinely measured samples on a qTOF instrument in ESI positive mode were performed forming a valproic acid positive group (determined by an additional targeted valproic acid method) and a negative reference group. The authors were able to identify eight potential (indirect) targets for valproic acid (Mollerup et al., 2019).

### Metabolomics for Biomarker Search of Acute Drug Intake or Manipulation

Application of alternative screening methods not directly targeting the analyte's or its metabolite's chemical structures but e.g., aim on certain endogenous biomarkers, seem to be a desirable approach that would facilitate the complex analytical scenario to detect NPS or chemical adulteration. Different strategies have been applied so far and are summarized in Tables 2, 3: (a) search for analytical, endogenous markers after a certain stimulus such as drug intake; (b) finding markers which derive from a common drug preparation, e.g., herbals used for “spice” products or non-physiological ingredients of artificial urine products; (c) search for endogenous markers that can level out inter-individual variations; (d) search for markers of urine manipulation attempts; and (e) identification of stable/unchanged markers that can proof integrity of a urine sample. Overall, data on such approaches for actual NPS are scarce and only few studies are available on common DOAs. Such studies—particularly in humans require highly controlled conditions which are of course ethically restricted for illegal drugs in many countries.

**Table 2:** Summary of studies applying metabolomics for biomarker search of acute drug intake or manipulation.

**Parent compound**	**Changed endogenous metabolites**	**Experimental setup**	**Sample preparation**	**Analytical conditions**	**Data evaluation**	**Reference**
MA	5-oxoproline Saccharic acid Uracil 3-hydroxybutyrate Adipic acid Glucose Glucose-6-phosphate Fructose 1,6-bisphosphate TCA cycle intermediates	Untargeted Rat plasma and urine MA vs. control *n* = 6 per group	LLE Derivatization CH_3_-O-NH_2_ MSTFA	GC-HRMS CP-SIL 8 (30 m × 0.25 mm i.d., 0.25-um) He TOF CE-MS/MS FunCap-CE type S 50 mM NH_4_Ac (pH9) QTrap	MetAlign SIMCA-P +	Shima et al., 2011
GHB	glycolate succinate creatinine	Untargeted Human urine Controlled administration Before/after design *n* = 12 per group	Lyophilization	NMR Bruker AVANCE II 600 14.1 T 1D ^1^H NMR 2D ^1^H-^1^H COSY 2D ^1^H-^1^H TOCSY 2D ^1^H- ^13^C HSQC 2D ^1^H-^13^C HMBC	SIMCA 14 Metaboanalyst	Palomino-Schatzlein et al., 2017
GHB	b-citryl glutamic acid	Untargeted Human urine Random GHB and reference samples *n* = 3 GHB *n* = 100 reference		LC-HRMS Eclipse XDB C18 (150 × 4.6 mm, 5 um) H_2_O, 0.1% FA ACN, 0.1% FA ESI pos/neg-qTOF	Agilent Profinder Agilent Mass Profiler Professional R version 2.11.1	Piper et al., 2017
GHB	Glycolate Succinylcarnitine	Untargeted Human urine Placebo vs. GHB controlled, crossover *n* = 19 each Authentic samples *n* = 10 GHB *n* = 20 control	Dilution/filtration	LC-HRMS XSelect HSST RP-C18 (150 mm × 2.1 mm, 2.5 μm) 10 mM NH_4_COOH, 0.1% FA MeOH, 0.1% FA Merck SeQuant ZIC HILIC (150 mm × 2.1 mm, 3.5 μm) 25 mM NH_4_Ac, 0.1% HOAc ACN, 0.1% HOAc ESI pos/neg—qTOF Fullscan Additional run in MS/MS mode (DDA)	Progensis Qi Metaboanalyst 4.0	Steuer et al., 2018c
Synthetic cannabinoids/ herbal blends	Scopoletin *N,N-*bis (2-hydroxyethyl) dodecylamine	Untargeted Human saliva Tobacco vs. 6 different herbal mixtures *n* = 3 per group	PP	LC-HRMS CORTECS® C18 (100 × 2.1 mm, 2.7-μm) H_2_O, 0.01% FA MeOH, 0.01% FA ESI pos/qTOF	MassLynx XCMS in R EZinfo 2.0	Bijlsma et al., 2018

**Table 3:** Summary of studies applying metabolomics for biomarker search of urine manipulation attempts.

**Investigated matrix**	**Changed endogenous metabolites**	**Experimental setup**	**Sample preparation**	**Analytical conditions**	**Data evaluation**	**Reference**
Artificial urine	Urine integrity marker: Phenylalanine Tryptophan Propionyl-carnitine Butyryl-carnitine Isovaleryl-carnitine Hexanoyl-carnitine Heptanoyl-carnitine Octanoyl-carnitine Indoleacetylglutamine Phenylacetylglutamine Marker for artificial urine: Tetrapropylene glycol Pentapropylene glycol Hexapropylene glycol Heptapropylene glycol Octapropylene glycol Nonapropylene glycol Decapropylene glycol Undecapropylene glycol	Untargeted acquisition Targeted data evaluation Random human Urine *n* = 550	PP	LC-MS/MS EC100/3 Nucleoshell RP18plus (100 mm × 2.1 mm; 2.7 μm) 10 mM NH_4_COOH, 0.1% FA ACN, 0.1% FA ESI pos/Ion trap DDA MS^n^	TF ToxID 2.1.1 Library assisted identification	Kluge et al., 2018
Artificial urine	BIT ethylene glycols (E3G, E4G)	Untargeted Artificial urine products Artificial vs. authentic *n* = 8 Authentic urine samples *n* = 4,000	Dilution	LC-HRMS Waters ACQUITY® HSS C18 (150 × 2.1 mm, 1.8 μm) 5 mM NH_4_COOH ACN ESI pos/qTOF	Manual data comparison	Goggin et al., 2017
Marker for chemical urine adulteration	Acetylneuraminic acid dimethyllysine Dimethyluric acid Glutamine Histidine Methylhistidine Methyluric acid Trimethyllysine Uric acid 5-HO-isourate 5-Hydroxy-2-oxo-4-ureido-2,5-dihydro1H-imidazole-5-carboxylate Imidazole lactate Methylimidazole lactate	Untargeted Human urine Untreated vs. treated *n* = 10 each Targeted marker testing Authentic urine samples *n* = 100	PP	LC-HRMS XSelect HSST RP-C18 (150 mm × 2.1 mm; 2.5 μm) 10 mM NH4COOH, 0.1% FA MeOH, 0.1% FA ESI pos/qTOF Full scan DDA MS/MS	XCMS^Plus^ Metaboanalyst 3.0	Steuer et al., 2017 Steuer et al., 2018a,b

#### Search for Analytical, Endogenous Biomarkers of Drug Intake

**Figure 4:**
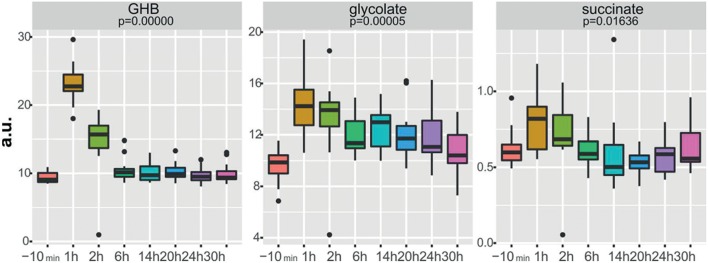
Boxplots of normalized relative concentrations of GHB, glycolate, and succinate at different time points after GHB-intake. *p*-values from ANOVA are indicated. Reprinted (adapted) with permission from Palomino-Schatzlein et al. (2017). Copyright 2017, American Chemical Society.

**Figure 5:**
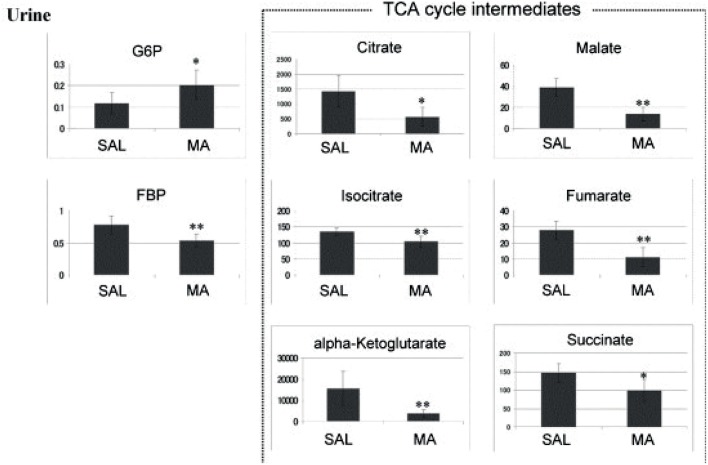
Anionic metabolites identified in a 0–24 h urine samples using CE-MS. *p < 0.05, **p < 0.01 methamphetamine (MA) vs. saline (SAL). Reprinted (adapted) with permission from Shima et al. (2011). Copyright 2011, Elsevier.

New strategies for GHB detection and differentiation between endogenous and exogenously consumed or administered GHB is still of high interest in forensics. Also, metabolomic studies were recently performed in order to find endogenous markers that might be able to prolong the detection window of GHB (Palomino-Schatzlein et al., 2017; Steuer et al., 2018c). Palomino-Schatzlein et al. used an NMR-based metabolomics approach to identify changes caused by GHB in a controlled administration study in human urine. As indicated by urine OPLS-DA analysis and S-plots derived from recorded ^1^H metabolic data gave an indication for highest influence on separation for GHB itself, glycolate and succinate. Glycolate as well as succinate have been previously associated with the metabolism of endogenous GHB. Further evaluation of the potential usefulness of succinate and glycolate as surrogate biomarkers of GHB intake was performed by quantitative ^1^H-NMR experiments, checking for time-related changes of their normalized relative concentrations (at −10 min and 1, 2, 6, 14, 20, 24, and 30 h postdose). As demonstrated in Figure 4, GHB and succinate concentrations were shown to drop to baseline levels already after 6 h post intake, while glycolate concentration declined at a much slower rate with small differences compared to baseline even after 24 h. In this context, glycolate has been discussed by the authors as a potential biomarker exceeding the window of detection of GHB itself (Palomino-Schatzlein et al., 2017). A similar study using HRMS based metabolomics on endogenous changes in urine after controlled GHB administration in humans was recently published by Steuer et al. Next to the identified conjugates of GHB with carnitine and amino acids, in accordance with the former study, significant changes in succinylcarnitine and glycolate could be observed in samples collected (only) 4.5 h after GHB intake. While significant differences (in controlled, paired samples, placebo vs. GHB intake) could be observed (Figure 3), the authors considered the observed increases in glycolate, succinate or succinylcarnitine as insufficient to provide reliable proof of GHB intake under highly variable inter-individual physiological conditions (Steuer et al., 2018c). Despite the fact, that other DOAs such as methamphetamine (MA) show better pharmacokinetic properties and can be easily measured over longer time frames in biological matrices, there is a certain interest in alternative (indirect) markers of their consumption. For instance, Shima et al. used an untargeted metabolomics approach with GC-HRMS for the analysis of rat plasma and urine. While their primary objective was to elucidate the underlying mechanism of several intoxication effects, they also evaluated the usefulness regarding indirect analytical detection of MA intoxication. The study proposed the following endogenous compounds to be considered as potential markers of MA intoxications: 5-oxoproline, saccharic acid, uracil, 3-hydroxybutyrate (3-HB), adipic acid, glucose, glucose 6-phosphate, fructose 1,6-bisphosphate, and tricarboxylic acid (TCA) cycle intermediates like fumarate (Shima et al., 2011). Typical changes observed in TCA cycle intermediates are exemplified in Figure 5. However, as already discussed above, it remains questionable whether or not these compounds will actually serve as sufficient discriminants in random urine samples. Furthermore, the changes in the identified endogenous compounds are most likely not specific for GHB or MA as will be in detail discussed under section Current Limitations and Discussion.

#### Search for Markers Which Derive From a Common Drug Preparation

**Figure 6:**
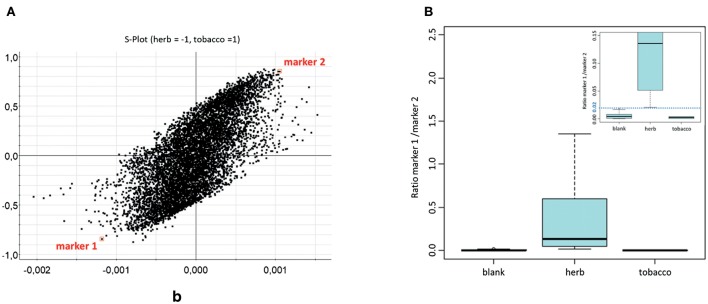
**(A)** Representation of all features from OPLS-DA, shown as S-plot. Marker features are indicated in squares. **(B)** Boxplot depicting peak area ratios between marker 1 and marker 2 in saliva samples; separated as blank, after herb smoking and after tobacco smoking (*n* = 24, 18, 6, respectively). Reprinted (adapted) with permission from Bijlsma et al. (2018). Copyright 2018, Springer.

Instead of focusing on the DOA or NPS itself, another promising approach might target common base products for drug preparations. Such a strategy was pursued in order to find markers for herbal mixtures, which act as the herbal base for “spice” products. Data were obtained with an innovative untargeted MS metabolomics approach in human saliva after smoking of six natural herbal components (*Canavalia maritima, Leonurus sibiricus, Althaea officinalis, Turnera diffusa, Verbascum Thapsus, and Calendula officinalis*). Combined with appropriate statistical analysis, two markers [scopoletin and *N,N*-bis(2-hydroxyethyl)dodecylamine] could be highlighted as indicated in the S-plot in Figure 6 and structurally elucidated. The ratio of marker 1 over marker 2 allowed the differentiation of non-smokers from herb consumers. Of course the current data still needs to be considered as preliminary, but nevertheless appears promising for further studies concerning time frames and changes, significance of the markers (e.g., their role in the herbal blends), method validation, etc. (Bijlsma et al., 2018).

Similar to the focus on herbal constituents instead of active ingredients, markers for artificial or “fake” urine products were evaluated. Goggin et al. aimed to discriminate fake urine samples from authentic ones through identification of unique substances present only in commercially available synthetic urine specimen and unexpected in biological samples. Benzisothiazolinone (BIT) and ethylene glycols [triethylene glycol (E3G), tetraethylene glycol (E4G)] were shown to identify a sample as being non-biological (Goggin et al., 2017). Other patterns of (high molecular) polypropylene glycols identical to those of purchased fake urine samples were identified by Kluge et al. (2018).

#### Search for Endogenous Biomarkers Leveling Out Inter-individual Variation

A large obstacle in interpretation, e.g., for GHB markers such as GHB-glucuronide or GHB-sulfate is their high inter-individual variation. This is one of the major issues that make distinction between GHB consumption vs. GHB control group via a defined cut-off level nearly impossible. In sports doping testing, analyte specific normalization is a well-established method, for instance for testosterone, to find concentrations above the expected physiological limits. Here, the testosterone concentration is evaluated not only as an absolute concentration level but additionally as the ratio to the epitestosterone concentration (Mareck et al., 2008). Similar to the strategies applied in doping testing, Piper et al. aimed to use an MS metabolomics-based approach to screen a reference population for possible endogenous compounds correlating significantly with GHB-glucuronide and GHB-sulfate changes to normalize their urinary concentrations (Piper et al., 2017). Beta-citryl glutamic acid was identified as the most promising candidate for normalization. The ratio between GHB-glucuronide and beta-citryl glutamic acid indicated high correlation in the extraction pattern for two tested chronical GHB consumers. On the other hand, a first-time GHB user provided a completely different profile (Piper et al., 2017). Further research on the suggested approach especially with higher numbers of GHB positives will be necessary prior to final evaluation.

#### Search for Markers of Urine Manipulation Attempts

An additional question addressed by metabolomics-like approaches was the identification of endogenous biomarkers or markers formed from those as indirect indicators for chemical urine adulteration. An ideal time- and cost-efficient workflow to test for urine adulteration—particularly for high throughput analyses—would allow integration of adulteration testing in the same analytical run applied for drug screening or quantification. Considering the chemical property of the adulterant to oxidize a drug and thereby cause massive decreases in sensitivity (until its potential un-detectability), oxidation of other (endogenous) urinary constituents appears likely as well. In contrast to classic discovery metabolomics, where changes in physiological pathways are evaluated, this approach aimed to identify changes caused by *in vitro* manipulation/oxidation of urine samples outside the body. Nevertheless, the applied workflows were exactly the same as in classical metabolome studies. In a first untargeted approach it was possible to identify several potential biomarkers exemplified for adulteration attempts with KNO_2_ using HRMS (Steuer et al., 2017). Further targeted metabolome studies utilizing a validated method for the selected markers provided promising results for uric acid (specificity 1.0, sensitivity 0.9) and two of its oxidation products, indolylacryloylglycine (specificity 0.9, sensitivity 1.0), and acetylneuramic acid as markers for KNO_2_ (Steuer et al., 2018a) and four other chemical adulterants (Steuer et al., 2018b).

#### Identification of Stable/Unchanged Markers

In contrast to classic metabolomics studies, a differing approach was conducted to identify stable markers in a large cohort of samples to differentiate treated or changed samples from control samples. For instance Goggin et al. and Kluge et al. defined and tested a number of endogenous urinary metabolites to accurately identify a tampered urine sample e.g., an artificial urine (Goggin et al., 2017; Kluge et al., 2018). For example, detection of less than six markers out of initially 10 present in authentic urine samples with a likelihood of >95% (phenylalanine, tryptophan, propionyl-carnitine, butyryl-carnitine, isovaleryl-carnitine, hexanoyl-carnitine, heptanoyl-carnitine, octanoyl-carnitine, indoleacetylglutamine, phenylacetylglutamine) could be considered as a hint for urine adulteration (Kluge et al., 2018).

### Metabolomics for Biomarker Search of Drug Addiction

Metabolomics combined with DOAs did not only focus on biomarkers indicating acute drug consumption but also on identification of hints for drug addiction, the severity of drug addiction or the interpretation of the severity of a given intoxication. While these studies certainly do not help in terms of new strategies for NPS detection, they can be useful in interpretation of clinical or forensic analytical results. Currently available literature focused on cocaine, crack, heroin, and MA. An overview of the analytical methods used and potential markers identified is given in Table 4.

**Table 4:** Summary of studies applying metabolomics for biomarker search of drug addiction.

**Parent compound**	**Changed endogenous metabolites**	**Involved biological pathways**	**Experimental setup**	**Sample prep**	**Analytical conditions**	**Data evaluation**	**Reference**
Cocaine	N-methylserotonin Guanine Hypoxanthine Anthranilate Xanthine	Tryptophan metabolism Purine metabolism	Targeted Human plasma Cocaine-dependent (*n* = 18) vs. healthy controls (*n* = 10)	PP	Electrochemical detection electrochemical array platform LCECA	R	Patkar et al., 2009
Crack	Lactate Long chain fatty acids acylated carnitines Histidine and tyrosine	Nutritial behavior	Untargeted Human serum Crack-dependent vs. control group *n* = 44 per group	Dilution	NMR ^1^H-NMR (1D, 600.173 MHz) T2-edited spectra of ^1^H NMR HSQC experiments for ^1^H–^13^C correlations		Costa et al., 2018
Heroin	myo-inositol-1-P threonate 9-z-hexadecenoic acid hydroxyproline		Untargeted Rat serum/urine Control vs. heroin-treated *n* = 6 per group	PP Derivatization CH_3_-O-NH_2_ MSTFA	GC-MS DB5-MS (10 m × 0.18 mm) He EI/TOF Fullscan	SIMCAP 11	Zheng et al., 2013
MA	Acute: Alanine Glycine Ornithine Asparagine Valine Isoleucine Leucine Serine Proline Threonine Methionine Citrulline Tryptophan Glutamine Glutamate Asparate Lysine Citrate 2-ketoglutarate Succinate Fumarate Malate Pyruvate Long term (5 days): Alanine Glycine Lysine Threonine Ornithine, hydroxyproline citrulline Fumarate Pyruvate Succinate Citrate 3-hydroxybutyrate 5-hydroxyindoleacetic 2 days after withdrawal Isoleucine Palmitic acid Creatinine Citrate 2-ketoglutarate Lactate	Energy metabolism Amino acid metabolism TCA cycle Lipids and free fatty acids	Untargeted Rat serum/urine Control vs. MA-treated *n* = 6 per group	PP Derivatization CH_3_-O-NH_2_ MSTFA	GC-MS RTx-5MS column (30 m × 0.25 mm) He EI Fullscan	SIMCA-P 13	Zheng et al., 2014

For example, Costa et al. performed ^1^H NMR-based metabolomics analysis of crack users' serum samples aiming to investigate whether drug dependency changes the endogenous profile and to further identify potential biomarkers that might be linked to brain dysfunction. The rationale of the study was the current lack of reliable diagnostic tools for crack dependency that at present mainly relies on self-reporting, medical history and physical examination. Early diagnosis should, however, result in better treatment outcomes. Serum samples of two groups were compared, crack users on the one hand against healthy individuals with similar age, gender and body mass index on the other hand. Differences were observed particularly in lactate, acylcarnitines, histidine, tyrosine, and phenylalanine which according to the author's opinion could be linked to altered brain functions. PLS-DA obtained 89.8% accuracy in differentiation of crack users from healthy controls. However, these observations must be considered cautiously due to confounding factors such as medical treatment of some crack users and the small sample size of drug users examined in general (Costa et al., 2018).

An untargeted metabolome analysis using GC-MS in rats receiving heroin for 10 days followed by a withdrawal of 4 days indicated increased myo-inositol-1 phosphate levels and decreased threonate concentrations in serum. In contrast to other biomarkers observed, these levels did not restore to baseline even after heroin withdrawal for 4 days. Therefore, these compounds were discussed as potential indicators of heroin abuse even when the consumer has been abstinent from heroin for some days. An even more sensitive marker would be the ratio of myo-inositol-1-phosphate to threonate. The drug morphine, which is also the main metabolite of heroin, interestingly did not result in the same changes as described for heroin itself. This, in the author's opinion, might potentially allow a differentiation of heroin addiction from morphine dependency (Zheng et al., 2013). However, so far, these findings have never been confirmed in larger (human) studies.

As already described in the Search for Analytical, Endogenous Biomarkers of Drug Intake, Shima et al. identified 5-oxoproline, saccharic acid, uracil, 3-HB, adipic acid, glucose, glucose 6-phosphate, fructose 1,6-bisphosphate, and TCA cycle intermediates like fumarate, as potential biomarkers helpful to assess the severity of MA-induced intoxications (Shima et al., 2011). A follow-up study on chronic MA intake and MA addiction to proof the initial findings to be specific for acute MA toxicity resulted in quite different results compared to previously found acute effects. As a conclusion, different metabolomic changes can be observed depending on the MA tolerance—either acute or chronic consumption. MA tolerance after chronic consumption could have resulted in several adaptations with no significant changes in the metabolite levels (Zaitsu et al., 2014).

### Metabolomics to Study Acute and Chronic Toxicity Mechanisms

The last and major group of currently available metabolome approaches linked to DOAs include studies primarily aiming to elucidate the mechanisms of drug action. While these studies were not designed to identify biomarkers, they might serve as a basis to further evaluate the general applicability of metabolome approaches for biomarker discovery. For example, they might provide first hints or estimates to assess the specificity for a particular DOA, the time frames of observed effects or confounding factors caused by other drugs or diseases. Observed metabolome changes, study design and analytical conditions of these studies are summarized in Table 5. A good summary can also be found in three recent review articles on DOAs and metabolomics (Dinis-Oliveira, 2014; Zaitsu et al., 2016; Ghanbari and Sumner, 2018). For a more detailed discussion on the mechanistic effects, the interested reader is referred to these works. The current review will just use results from the publications that appear helpful in the context of biomarker detection and evaluation of their general applicability.

**Table 5:** Summary of studies applying metabolomics to study acute and chronic toxicity mechanisms.

**Parent compound**	**Changed endogenous metabolites**	**Pathway**	**Experimental setup**	**Sample preparation**	**Analytical conditions**	**Data evaluation**	**Reference**
CBD	UDP-GlcNac Glutathione Oxidized glutathione Tyrosine fructose 1,6-diP Glycerol 3-phosphate Dihydroxyacetone-P Xanthine Succinic acid Arachidonic acid 5-methylcytidine PE(18:1(9Z)/0:0)	Glucose metabolism Antioxidant activity Catecholamines biosynthesis Glycolysis TCA cycle	Untargeted Rat brain Placebo vs. CBD *n* = 5 per group	Homogenization SPE for phospholipid removal	LC-HRMS Poroshell 120 EC-C18 (100 × 3.0 mm, 2.7 μm) H_2_O, 0.1% FA ACN, 0.1% FA ESI pos/neg-qTOF Fullscan DDA auto MS/MS function	XCMS online Metaboanalyst 3.0	Citti et al., 2018
Cocaine	Threonine Cystine Spermidine *N*-propylamine,		Untargeted Rat plasma/ urine control vs. drug *n* = 6 per group	LLE Derivatization CH_3_-O-NH_2_ MSTFA	GC-HRMS CP-SIL 8 column (30 m × 0.25 mm i.d., 0.25-um) He TOF	Built-in software SIMCA-P + 13	Zaitsu et al., 2014
Cocaine/EtOH	Argininosuccinic acid Cystathionine *N*-acetyl-l-lysine Methionine Carnosine Spermidine Serotonin	Amino acid metabolism Oxidative stress	Untargeted Rat plasma Control (*n* = 9) vs. cocaine (*n* = 8) vs. EtOH (*n* = 10) vs. cocaine/EtOH (*n* = 10)	PP Filtration FMOC	LC-HRMS C18 Ascentis Express (100 × 2.1 mm 2.7 um) H_2_O, 0.1% FA ACN, 0.1% FA ESI pos/qTOF Full scan MS/MS (DDA)	XCMS in R SIMCA R	Sanchez-Lopez et al., 2017
GHB	Acute: Glycolysis metabolites glucose 6P Fructose-6P Fructose 1,6-diP glucose 1,6-diP Glycolate 2-hydroxyglutarate Glucose Pyruvate Acetyl-CoA Citrate Malate Lysolipids Long-chain acylcarnitines 3-HO-butyrate Chronic: 2-hydroxyglutarate Linoleoglycerophospho- cholines		Untargeted Mice brain/liver Placebo vs. GHB *n* = 8 per group	Not described	Not described	Not described	Luca et al., 2015
	Long-chain acylcarnitines 3-HO-butyrate S-adenosyl homocysteine Glutathione Riboflavin ↓ Reduced glutathione Inosine 5'P Guanosine 5'P Cytidine 5'P *N*-acetylmethionine ↑ Fatty acids Omega-3 fatty acids						
MAM 2201	Malic acid Succinic acid Glutamic acid L-phenylalanine	Energy metabolism Glutamatergic nervous system	Untargeted Rat cerebrum Control vs. MAM 2201 (low and high concentration) *n* = 5 per group	LLE Derivatization CH_3_-O-NH_2_ MSTFA	GC-HRMS TOF DB-5 capillary column (30 m × 0.250 mm; 1 μm) He EI/TOF Fullscan SRM	SIMCA-P+13	Zaitsu et al., 2015
MDMA	5HT Carnitine Choline	Energy regulation	Targeted Rat heart tissue Control vs. MDMA *n* = 4–8 per group	Dilution	NMR High resolution magic angle spinning ^1^H-NRS 1-D CPMG pulse sequence HPLC C18-RP column ESA MD-TM mobile phase Coulometric detection (ESA 5011A dual electrode cell)	GraphPad Prism 4 SPSS v16.0	Perrine et al., 2009
MDMA	AMP Adenosine Inosine *S*-adenosyl-L- Homocysteine Hexanoylcarnitine 2-octenoylcarnitine Decanoylcarnitine 9-decenoylcarnitine Dodecanoylcarnitine Tetradecenoylcarnitine Uric acid Tryptophan Thiomorpholine 3-carboxylate	Energy metabolism Serotonin synthesis	Untargeted Retrospective Human whole blood MDMA positive vs. control *n* = 74 per group	PP	LC-HRMS ACQUITY BEH C18 (100 mm × 2.1 mm, 1.7 μm) H_2_O, 0.1% FA ACN, 0.1% FA ESI pos /qTOF Fullscan bbCID undirected fragmentation.	XCMS in R SIMCA version 13	Nielsen et al., 2016
	LysoPC (16:0) LysoPC (17:0) LysoPC (18:1) Glycerol	Energy metabolism Serotonin synthesis	Untargeted Retrospective Human whole blood MDMA positive vs. control *n* = 74 per group				
MDMA	Spinganine Arginine Asparagine Glycine Glutamine Histidine Methionine Met-SO/Met Trimethylglycine Tryptophan Tyrosine Biliverdin Carnitine Propionylcarnitine Hydroxy-eicosatetraenoic acid Oleamide Docosenamide Hexadecanoyl glycerol Glycerol stearate PCs Dihydroxy-eicosatetraenoic acid Octadecadienoic acid LysoPCs Calcitriol Cortisol Pregnenolone sulfate	Energy metabolism Steroid metabolism Inflammation Oxidative stress	Targeted Untargeted Human plasma Controlled administration Placebo vs. MDMA Crossover design *n* = 16 per group		LC-(FIA)-MS/MS Biocrates Kit (Biocrates, 2010) ESI pos/neg - qTrap MRM LC-HRMS XSelect HSST RP-C18 (150 mm × 2.1 mm, 2.5 μm) 10 mM NH_4_COOH, 0.1% FA MeOH, 0.1% FA Merck SeQuant ZIC HILIC (150 mm × 2.1 mm, 3.5 μm) 25 mM NH_4_Ac, 0.1% HOAc ACN, 0.1% HOAc ESI pos/neg - qTOF Fullscan Additional run in MS/MS mode (DDA)	Progensis Qi Metaboanalyst 4.0	Boxler et al., 2017, 2018b
MA	Acute Lactate Malate 2-Hydroxyglutarate Succinate Tryptophan Fumarate Fructose Sorbitol	Acute Alpha linolenic acid and linoleic acid metabolism Energy metabolism TCA cycle Mitochondrial electron transport chain Repeated Oxidation of branched chain fatty acids	Untargeted Mouse brain−8 different mice strains Acute Control (*n* = 6 per strain) vs. MA (*n* = 3 per strain) Repeated Control (*n* = 3 per strain) vs. MA (*n* = 6 per strain)	Homogenization BSTFA for GC-MS	LC-MS/MS ESI pos/neg —Ion trap—FT-ICR Fullscan MS/MS (DDA) GC-MS 5% phenyl column EI Fullscan	In-house software (Metabolon Inc) R/Stata Metaboanalyst	McClay et al., 2013
	Putrescine Ergothioneine Phosphocholine Tyrosine Serotonin 3-dehydrocarnitine						
MA	N-propylamine Lauric acid Lactose Spermidine Stearic acid		Untargeted Rat plasma/ urine Control vs. drug *n* = 6 each	LLE Derivatization CH_3_-O-NH_2_ MSTFA	GC-HRMS CP-SIL 8 column (30 m × 0.25 mm i.d., 0.25-um) He TOF	Built-in software SIMCA-P+13	Zaitsu et al., 2014
Opioids Methadone	GSH/GSSG Tocopherol Guanine Xanthosine Guanosine hypoxanthine *N*-methylserotonin	Antioxidant activity Purine metabolism	Targeted Human plasma Opioid-dependent (*n* = 14) vs. healthy controls (*n* = 10)	PP	Electrochemical detection Electrochemical array platform LCECA	R	Mannelli et al., 2009
Opioids Morphine	Glutamate Glutamine GABA Succinic acid semialdehyde Phosphocholine Acetylcystein Lactic acid Creatine Methionine Taurine α-ketoglutarate Succinate Homocysteic acid	Neurotransmitter metabolism Oxidative stress	Untargeted Rat brain Control vs. morphine vs. Morphine/methadone vs. Morphine/clonidine *n* = 6 per group	Homogenization LLE Lyophilization	NMR ^1^H-NMR 1D CPMG pulse sequence	MestRe-c2.3 SIMCA	Hu et al., 2012
Opioids Heroin	Aspartate Myo-inositol-1P docosahexaenoic acid Hydroxyproline 5-hydroxytryptamine 9-(z)-hexadecenoic acid Palmitic acid Threonate 5-hydroxy indoleacetate Citrate Tryptophan Hydroxyproline	TCA cycle Fatty acid metabolism	Untargeted Rat serum/urine Control vs. heroin *n* = 6 per group	PP Derivatization CH_3_-O-NH_2_ MSTFA	GC-MS DB5-MS (10 m × 0.18 mm) He EI/TOF Fullscan	SIMCAP 11	Zheng et al., 2013
	Aspartate Fumarate Glycine Lactate	TCA cycle Fatty acid metabolism					
Opioids Morphine	3-hydroxybutyric acid Tryptophan Cystine N-propylamine 2-ketoglutaric acid, fumaric acid Malic acid Threonine Glutamic acid Isoleucine Valine Aspartic acid Oxamic acid 2-aminoethanol indoxyl sulfate creatinine	TCA cycle Energy metabolism	Untargeted Rat plasma/ urine control vs. drug *n* = 6 per group	LLE Derivatization CH_3_-O-NH_2_ MSTFA	GC-HRMS CP-SIL 8 column (30 m × 0.25 mm i.d., 0.25-um) He TOF	Built-in software SIMCA-P+13	Zaitsu et al., 2014
Opioids Heroin	Choline Phosphocholine Glycerol Glutamine Phenylalanine Fatty acids Glucose Pyruvate Fumarate Lactate 3-hydroxybutyrate Acetoacetate Acetone	TCA cycle Energy metabolism Phospholipid metabolism, Keto body metabolism Central carbon metabolisms Neurotransmitter	Untargeted Rat serum Heroin (*n* = 7) vs. control (*n* = 8)		NMR ^1^H-NMR 1D CPMG pulse sequence	SIMICA-P 11.0 KEGG pathway analysis.	Ning et al., 2018

As can be seen in Table 5, certain changes in endogenous compounds are detected for all studied DOAs. However, the observed changes, albeit statistically significant, appear rather small (Figure 5), particularly when they should allow for differentiation in a non-controlled setting such as drug testing of random subjects. Furthermore, very often similar compounds or in general the same pathways e.g., the energy metabolism or the TCA cycle, are affected. From a pharmacological point of view this is not surprising. As stated e.g., by Ning et al. neuronal activity is extremely energy demanding, and the brain energy supply requires oxidative metabolism of glucose in mitochondria and demands lactic acid from glycolytic processes (Ning et al., 2018). However, albeit e.g., MA and GHB act at different pharmacological targets both were shown to influence succinate concentrations (Zheng et al., 2014; Palomino-Schatzlein et al., 2017). It remains to be determined whether or not the observed changes might ever be able to specifically indicate consumption of a particular DOA/NPS or substance groups or drug use in general. Most likely, changes of single endogenous metabolites will be too unspecific for a certain drug or even drug class. Evaluating fingerprints or changes in particular metabolic pathways appear more promising, but currently lack sufficient studies to support or reject this hypothesis.

Also, other confounding factors need to be considered such as underlying diseases. For example, Mannelli et al. found elevated levels of *N*-methylserotonin in plasma of human opioid abusers. *N-*methylserotonin is an analog to serotonin and similar to the derivative bufotenine, both are known for their hallucinogenic and psychotropic effects. However, also in terms of psychiatric disorders associated with hallucinations and altered perceptions elevated levels of e.g., serotonin can be found (Takeda, 1994; Takeda et al., 1995; Mannelli et al., 2009).

The kind and state of drug consumption of course also influences the outcome on the metabolome. Addiction, withdrawal and relapse can also change the metabolome in partly similar ways as acute drug consumption (Zheng et al., 2013, 2014). In some studies, however, drug addiction/chronic drug use may change and/or eliminate the observed effects as e.g., shown for MA (Zaitsu et al., 2014) (see Metabolomics for Biomarker Search of Drug Addiction).

Well-designed controlled studies in rats can give first insights into the duration of the metabolome effects. For example, multivariate analysis (PLS-DA plots) showed that after withdrawal from MA for 2 days (after initial 5 days intake) metabolite values of those rats clustered close to the values of the control group, yet not overlapping with the control data. This is suggestive of an efficient restoration of urine metabolites to baseline levels after withdrawal (Zheng et al., 2014). In contrast to MA, metabolic changes induced by heroin recovered more slowly and were more pronounced (Zheng et al., 2013, 2014). For instance, heroin withdrawal for 4 days did neither restore elevated serum myo-inositol-1phosphate levels nor decreased serum threonate to values prior to heroin consumption. Similar findings were observed for 9-(z) hexadecenoic acid in serum and hydroxyproline in urine with little effect of heroin withdrawal on their concentration levels (Zheng et al., 2013).

The currently available knowledge is of course too preliminary to draw any final conclusion. It definitely needs to be considered that all studies mentioned here were performed for a totally different aim. An overall, critical discussion on these studies as well as the few studies performed for actual biomarker research will be provided in section Current Limitations and Discussion.

## Current Limitations and Discussion

Metabolomics to identify potential biomarkers that can act as indirect indicators of drug consumption would be an interesting approach to tackle the problem of the increasing number of new drugs flooding the market. At present, only few studies have been performed, nevertheless showing promising first results to identify analytical biomarkers by metabolomics-related techniques. However, far more studies will be necessary for a final conclusion on the general suitability of metabolomics in drug testing. From the current state of knowledge several critical points and limitations can be deduced that shall be discussed in the following and might help to effectively plan further studies.

From the analytical point of view, untargeted analysis is considered most promising to identify a priori unknown metabolites and pathways which are reflected in the current literature (see, Tables 1–5). While these approaches cover a broad range of compounds allowing identification of a number of different pathways, they lack sensitivity particularly for low abundant metabolites. Up to now, targeted studies considering first results from untargeted analysis are rarely performed but might help to identify more reliable biomarkers as shown in recent studies by Olesti et al. They applied targeted metabolome approaches mainly focusing on neurotransmitters to successfully predict the pharmacological profile of NPS (Olesti et al., 2019a,b). Also improvement of statistical methods, commercial or customized software solution including e.g., deep-learning approaches (Asakura et al., 2018; Grapov et al., 2018) will improve marker finding in the future.

Up to now many studies were performed with rather small sample sizes and lack comparability in terms of used species, matrices, experimental set-up, time-frames, etc. This limits common conclusions of the available results. For example for MA, contradictory results were obtained by two independent studies. While associations with the TCA cycle (fumarate, malate, succinate) was found in both studies, one study found reduced levels in comparison to controls and interpreted this as a reduction in energy metabolism (Shima et al., 2014), whereas in the second study levels were found to be increased (McClay et al., 2013). Most likely these differences can be related to different study set-ups in terms of administered doses and/or time and duration of administration and subsequent sample collection. As highly controlled conditions are mandatory to actually identify potential biomarkers, the majority of the current research was done in animal models. Data on how the results transfer to humans are still missing.

Finally, the metabolome is highly variable which means that many confounding factors, e.g., from food or exercise, but also from other drugs, prescription drugs and underlying diseases will need to be evaluated. At present, there are several untargeted metabolome studies but follow-up studies actually proving the suitability of the proposed markers under inter-individual variations in routine work in terms of sensitivity/specificity are largely missing. However, if actually performed, general suitability of markers identified in global approaches could be confirmed (Steuer et al., 2018a; Mollerup et al., 2019).

## Conclusion

In conclusion, metabolomic approaches possess, in general, great potential for detection of biomarkers indicating drug consumption. It is also an interesting approach in drug metabolism research (xenometabolomics)—particularly for seldom or unusual metabolites. Changes observed so far on the endogenous level currently appear rather small and partly unspecific and might be insufficient on the level of single markers to reliably prove drug consumption. But, most importantly, more studies, including more sensitive targeted follow-up analyses as well as multivariate statistical models or deep-learning approaches are strongly needed to fully explore the potential of omics science in DOA testing. Future studies need to be highly controlled with reasonable sample sizes and require, in the authors opinion, targeted, proof-of-concept studies including the evaluation of confounding factors, and sensitivity/specificity assessment subsequent to the initial global profiling approaches. Progress in analytical techniques as well as in deep learning approaches will facilitate the more and more complex data evaluation necessary for studies including huge numbers of analytes and samples.

## Author Contributions

AS performed the literature research and wrote the manuscript. LB and TK wrote the manuscript.

### Conflict of Interest Statement

The authors declare that the research was conducted in the absence of any commercial or financial relationships that could be construed as a potential conflict of interest.

## Abbreviations

^13^C: carbon-13
1D: one-dimensional
^1^H: hydrogen
2D: two-dimensional
3-HB: 3-hydroxybutyrate
5HT: 5-hydroxytryptamine
CAN: acetonitrile
AM2201: [1-(5-fluoropentyl)-1H-indol-3-yl]-1-naphthalenyl-methanone
AMP: adenosine monophosphate
ANOVA: analysis of variance
bbCID: broadband collision-induced dissociation
BIT: benzisothiazolinone
CBD: cannabidiol
CE: capillary electrophoresis
CH_3_-O-NH_2_: methoxyamine
CoA: coenzyme A
COSY: correlation spectroscopy
CPMG: Carr-Purcell-Meiboom-Gill
DDA: data-dependent acquisition
DIA: data-independent acquisition
diP: diphosphate
DOA: drug of abuse
E3G: triethylene glycol
E4G: tetraethylene glycol
EI: electron ionization
EMCDDA: European Monitoring Centre for Drugs and Drug Addiction
ESI: electrospray ionization
EtOH: ethanol
FA: formic acid
FIA: flow injection analysis
FMOC: fluorenylmethyloxycarbonyl
FT: Fourier transformation
GABA: gamma-aminobutyric acid
GC: gas chromatography
GHB: gamma-hydroxybutyric acid
GSH: glutathione
GSSG: glutathione disulfide
H_2_O_2_: hydrogen peroxide
He: helium
HILIC: hydrophilic liquid interaction chromatrography
HMBC: heteronuclear multiple bond correlation
HMDB: Human Metabolome Database
HO: hydroxy
HOAc: acetic acid
HPLC: high performance liquid chromatrography
HR: high resolution
HSQC: heteronuclear single-quantum coherence
HSST: high strength silica technology
I_2_: iodine
IA: immunoassay
ICR: ion cyclotron resonance
IS: internal standard
JWH-018: (1-pentyl-1H-indol-3-yl)-1-naphthalenyl-methanone
KEGG: Kyoto Encyclopedia of Genes and Genomes
KNO_2_: potassium nitrite
LC: liquid chromatography
LCECA: liquid chromatography electrochemical array
LLE: liquid-liquid extraction
MA: methamphetamine
MAM 2201: [1-(5-fluoropentyl)-1H-indol-3-yl](4-methyl-1-naphthalenyl)-methanone
MDMA, 3: 4-methylenedioxymethamphetamine
MeOH: methanol
Met: methionine
Met-SO: methionine sulfoxide
MRM: multiple reaction monitoring
MS: mass spectrometry
MSTFA: *N*-methyl-*N*-(trimethylsilyl)trifluoroacetamide
m/z: mass-to-charge ratio
NaOCl: sodium hypochlorite
NH_4_Ac: ammonium ethanoate
NH_4_COOH: ammonium formate
NIST: National Institute of Standards and Technology
NMR: nuclear magnetic resonance
NPS: new psychoactive substance
NRS: nuclear resonant scattering
OPLS-DA: orthogonal partial least-square–discriminant analysis
P: phosphate
PC: phosphatidylcholine
PCA: principle component analysis
PCC: pyridinium chlorochromate
PLS-DA: partial least-square– discriminant analysis
PMI: postmortem interval
PP: protein precipitation
QC: quality control
qTOF: quadrupole time-of-flight
RP: reversed-phase
SPE: solid phase extraction
SRM: selected reaction monitoring
SST: system suitability test
TCA: tricarboxylic acid
TOCSY: total correlation spectroscopy
TOF: time-of-flight
UDP-GlcNac: uridine diphosphate-N-acetylglucosamine
UNODC: United Nations Office on Drugs and Crimes
US: United States of America
VIP: variable importance in projection
ZIC: zwitterionic.
